# Retrospective analysis of radiological investigation of surgically excised head and neck lipomas

**DOI:** 10.1007/s00405-024-08672-3

**Published:** 2024-05-13

**Authors:** Amy L. Schranz, Fiona Riordan, Roisin Dolan, Catriona Lawlor, Colin Morrison, Gary O’Toole, Ronan Killeen, Graeme McNeill, Rory O’Donohoe, Tom Moran, Fergal O’Duffy

**Affiliations:** 1 Graduate Entry Medicine, University College Dublin, Dublin, Ireland; 2 Otorhinolaryngology/Head and Neck Surgery, St. Vincent’s University Hospital, Dublin, Ireland; 3 Plastic, Reconstructive and Aesthetic Surgery, St. Vincent’s University Hospital, Dublin, Ireland; 4 Orthopaedics Department, St. Vincent’s University Hospital, Dublin, Ireland; 5 Radiology Department, St. Vincent’s University Hospital, Dublin, Ireland; 6 School of Medicine, University College Dublin, Dublin, Ireland

**Keywords:** Head and neck, Lipoma, Liposarcoma, Head and neck imaging, Radiology

## Abstract

**Purpose:**

Differentiating benign lipomas from malignant causes is challenging and preoperative investigative guidelines are not well-defined. The purpose of this study was to retrospectively identify cases of head and neck lipomas that were surgically resected over a 5-year period and to identify the radiological modality chosen and features discussed in the final report. Multidisciplinary outcomes and pathology reports were examined with a view to identifying high risk features of a lipoma to aid in future risk stratification.

**Methods:**

Retrospective chart review of pathology characteristics, radiological features (modality, size, calcifications, septations, globular/nodular foci), multidisciplinary discussion and history of presenting complaint was performed.

**Results:**

Two liposarcomas and 138 lipomas were identified. Twenty-two percent of all lipomas received radiological investigation. Twenty-two percent of imaging referrals were possibly inappropriate. Furthermore, radiological features suggestive of malignancy were not present in the final radiology report, *X*^2^ = 28.8, *p* < 0.0001.

**Conclusion:**

As expected, the incidence of liposarcoma is low. There is limited awareness of radiology referral guidelines superimposed with a tendency to over-investigate lipomas. Furthermore, radiological features suggestive of malignancy were inconsistently reported on and not documented in multidisciplinary discussions. Therefore, we propose a multidisciplinary checklist for referring physicians and radiologists to aid in diagnostic work-up.

## Introduction

The World Health Organization (WHO) classification groups soft tissue tumors into eleven categories, and further divides these categories into benign, intermediate, and malignant [1, 2]. The most common category of soft tissue tumors is the *Adipocyte.* A lipoma is a benign adipocytic soft tissue tumour which is typically well-circumscribed with various histopathological subtypes, such as angiolipoma, fibrolipoma, and spindle cell lipoma [2]. Whereas liposarcoma is a malignant adipocytic soft tissue tumor and can be differentiated from its benign counterpart by a positive MDM2 amplification on fluorescence in situ hybridization (FISH) [3]. However, differentiating benign from malignant adipocytic soft tissue tumors can be challenging, especially when faced with an atypical lipomatous tumor/well-differentiated liposarcoma, an intermediate entity that also possesses the MDM2 amplification and is locally aggressive. Additionally, intramuscular lipomas, although benign, can be poorly circumscribed and infiltrative [4]. Furthermore, atypical spindle cell/pleomorphic lipomatous tumor (ASPLT) is a new addition to the WHO classification of benign adipocytic neoplasms. ASPLT are often larger and are characterized by ill-defined tumor margins and a mixed proportion of atypical spindle cells, adipocytes, lipoblasts, and other histology [1, 5]. Given the heterogeneity of these benign and malignant entities diagnostic workup can be challenging. Management and treatment algorithm has traditionally been based on small retrospective case series rather than from large clinical trials.

Head and neck liposarcoma accounts for less than 5% of all liposarcoma [6], while a lipoma has a reported prevalence of 13–25% in the head and neck of adults [7]. It has been long appreciated that while lipomas below the clavicle are more common in female patients, lipomas found in the head and neck region are more common in males [8]. Head and neck lipomas are typically superficial, while deep-seated lipomas are rarer and tend to present when they are clinically larger [8]. El-Monem et al. reviewed patient demographics and clinical presentations of 26 head and neck lipomas over a three-year period. Lipomas were predominantly found in males (62.5%) and presented predominantly in the posterior subcutaneous neck (87%). Lipomas presented clinically as a slow-growing, painless neck swelling that had been present for 6 months to 7 years prior to surgical excision. On physical exam lipomas were a non-painful, round, mobile mass with a characteristic soft, doughy feel. Preoperatively, most lipomas were imaged using CT (*n* = 14), followed by US (*n* = 4), then MRI (*n* = 3). Additionally, Kim and Yang [9] reported two cases of lipoma unusually located in the parotid and sternocleidomastoid muscle and concluded that lipoma should be included in the differential of tumors involving the parotid gland. More recently, Najaf et al. reported 3 cases of symptomatic cervical lipomas, which highlighted the anatomical complexity of the neck and the mass effect that a benign lipoma can have [10]. For a full review of clinical, histological, and cytogenetic features of lipoma tumours in the head and neck, see de Bree et al. [6]. For a review of imaging features of all soft tissue tumours of the head and neck, we guide the reader to detailed pictorial reviews [7, 11, 12].

Although not specific to the head and neck, Kransdorf et al. reviewed the radiological features of CT and MRI that distinguished a lipoma from a well-differentiated liposarcoma. Thirty-five lipomas and 25 well-differentiated liposarcomas were retrospectively reviewed, assessing images for adipose tissue content, nonfatty components, and signal intensity and tissue attenuation. The authors concluded that features suggestive of malignancy included increased patient age, large lesion size, presence of thick septa (> 2 mm), nodule and/or globular areas, and decreased percentage of fat composition (< 75%)[13]. Furthermore, the study found male sex, thick septa, and associated non-adipose masses to increase the likelihood of malignancy by 13, 9, and 32-fold, respectively. Taken altogether, risk factors for malignancy include age > 55 years, male sex, previous malignancy at same site and mass > 5 cm[4, 11, 13]; while radiological features suggestive of malignancy include low percent fat containing lesion (< 75%), calcifications, thick septa > 2 mm and non-lipomatous nodular or globular foci [7, 11, 13].

To ensure that the most appropriate radiological investigation is performed, referral guidelines have been developed. The European Society of Radiology (ESR) developed the referral guidelines, *ESR iGuide*, based on the American College of Radiology (ACR) Appropriateness Criteria [14]. Similarly, the iRefer guidelines are produced by the Royal College of Radiologists in the UK. These guidelines are evidence-based and implement rating scales to help referrers choose the most appropriate imaging modality for a clinical problem. However, there are no specific referral guidelines for suspected head and neck lipoma. Instead, head and neck lipoma fall under musculoskeletal soft tissue tumour guidelines, which were most recently revised by the European Society of Musculoskeletal Radiology (ESSR) in 2015 [15]. These guidelines outline the patient history and clinical features that should be available to the radiologist, the recommended imaging modalities, as well as technical specifications and the criteria of what should be included in the radiology report. Briefly, the recommended first line investigation for patients with a suspected soft tissue tumour is ultrasound. However, if there is a clinical suspicion of malignancy, then a primary MRI should be considered. Additionally, the referrer should proceed to MRI following US if the tumour is below fascia, size > 5 cm, any reasonable chance of being malignant or any sonographic doubt/not completely accessible by US. MRI is the imaging method with the best soft tissue contrast, while radiography or CT can be complementary to show calcification or ossification and bone involvement. However, utilization of these guidelines remains poor. For example, Weiss and Colleagues (2021) investigated whether recommended MRI report elements were included in compliance with ESSR guidelines and found frequent deviations from standard protocol [16]. The study also found 32% of sarcoma MRI reports had a misinterpretation of the masses as benign. Additionally, a later study from the same group compared sarcoma radiology reports before and after ESSR guidelines in 2015 and found no improvement in the reports [17]. While these studies have assessed the utilization of ESSR Guidelines on malignant cases, no studies to date have assessed benign entities such as lipoma. Therefore, the objectives of this audit were to retrospectively identify cases of head and neck lipomas that were surgically resected over a 5-year period and to identify the radiological modality chosen, features discussed in the final report, and to compare management to the ESSR guidelines.

## Methods

### Data collection

**Fig. 1:**
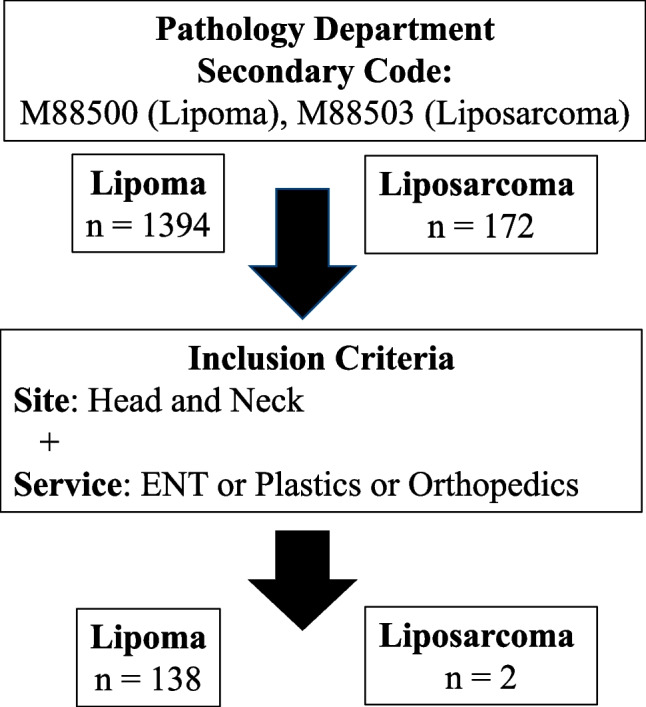
Data collection methodology

Approval was given for this study by The Group Clinical Audit Committee at our institution. Pathology records were queried for all patients with a Systemized Nomenclature of Medicine Clinical Terms (SNOMED CT) code M88500 (lipoma) and M88503 (liposarcoma) from January 2016 to December 2021 (Fig. 1). The query was further limited to head and neck patients treated by ENT, Plastics, or Orthopedic Surgeons. Retrospective chart review of age, sex, pathology characteristics (site, size, immunohistochemistry), radiological features (modality, size, calcifications, septations, globular/nodular foci, report conclusion), and MDT discussion was performed. Each lipoma case was assessed for risk factors for malignancy (age > 55, male sex, previous malignancy at same site, mass > 5 cm), and radiological features suggestive of malignancy (low percent fat containing lesion, calcifications, thick septa > 2 mm, non-lipomatous nodular or globular foci). Radiological features were identified as present, absent, or not discussed in the final radiology report.

### Statistical analysis

All statistical analyses were performed in GraphPad Prism, version 9.0 for Mac OS (GraphPad Software, San Diego California USA). Patient demographics between ENT and Plastic services were compared using an unpaired Students *t*-test. A Fisher’s Exact Test was used to examine lipoma site between ENT and Plastic services and examine lipoma management in patients with and without risk factors (size > 5 cm, male sex, age > 55, previous mass at same site). A Chi Square test was used to examine radiological features that were and were not discussed in the radiology report. An alpha value of 0.05 was used for all statistical tests.

## Results

### Demographics

From January 2016 to December 2021, *n* = 2 head and neck liposarcomas were identified in two patients, and *n* = 138 head and neck lipomas were identified in 136 patients. The final pathology report identified 129 benign lipomas (93%; two of which were recurrent lipomas), four benign fibrolipomas, three benign spindle cell lipomas, one intramuscular lipoma and one benign lipoma possible spindle cell/angiolipoma. Patient demographics can be found in Table 1. No oncological resections were identified in any of the lipoma cases, 10 cases were discussed at MDT, and 31 cases had imaging prior to local resection. Twenty-seven lipomas were excised through ENT, and 111 were excised through Plastics. When comparing both services, patients referred through ENT were on average older (Mean $\pm$ Standard Deviation; 54 $\pm$ 11) than patients referred through plastics (48 $\pm$ 12), *p* = 0.008. There were no significant differences in patient sex, or size of lipomas.

**Table 1:** Patient demographics

	Lipoma (*n* = 138)
Patients (*n*)	136
Sex, % M(F)	70(30)
Age, Ave $\pm$ SD	49 $\pm$ 12
Size, mm Ave $\pm$ SD	32 $\pm$ 25
Most common site (%)	Forehead (39) Posterior neck (20) Scalp (14) Anterior neck (12)

### Clinical risk factors for malignancy

Patients presenting with a soft tissue mass $\ge$ 5 cm were more likely to receive radiological investigation (OR 2.7 [1.1–6.7], *p* = 0.04), but not more likely to receive MDT discussion (*p* > 0.05). No other risk factors were significant when looking at radiological investigation or MDT discussion (age > 55 years, male sex, previous mass at same site). Patients presenting with 0 or 1 risk factor were not more likely to receive radiological investigation or MDT discussion compared to patients with 2–3 risk factors, and no patients had all four risk factors documented.

### Radiological investigation

Regions of the head and neck that received the most radiological investigation included the anterior neck (percent of region imaged = 76%) and supraclavicular region (67%), followed be the face (33%), posterior neck (24%), scalp (12%) and forehead (5%).

ENT services were more likely to receive referrals for lipomas in the neck (anterior and posterior) versus the head (e.g., forehead and scalp), odds ratio = 13.8 [4.7–35.3], *p* < 0.0001. Patients referred through ENT services were more likely to receive radiological investigation (73% versus 11% through Plastics), odds ratio = 22.3 [7.9–59.4], *p* < 0.0001.

Twenty-two percent of all lipomas received radiological investigation, as outlined in Table 2. Of these lipomas, 84% were initially investigated with ultrasound, while 16% were initially investigated with cross-sectional imaging (MRI or CT). Lipomas initially investigated with cross-sectional imaging did not have more risk factors for malignancy compared to those investigated initially with ultrasound (*p* > 0.05, Fisher’s Exact). Four patients received an MRI 1st line, and the indications provided were for a slowly enlarging mass of the posterior neck over 5 years (*n* = 1), painless mass of posterior neck (*n* = 1), occipital mass (*n* = 1) and one indication was not available for retrospective review. One patient was initially investigated with CT and had no indication provided for the scan, four patients had a second line CT following an US scan and the indications given were for further characterization of the soft tissue mass (*n* = 2), query liposarcoma (*n* = 1), and for a recurrent submandibular mass (*n* = 1). Of the five patients that received a CT scan, none had a documented contraindication to MRI. Taken all together, 22% of imaging referrals were inappropriate (9 of 41 referrals).

**Table 2:** Radiology ordering behaviour

Radiological investigation	# Risk factors^a^ for malignancy (%)^b^				Notes
	0	1	2	3	
Ultrasound (*n* = 26)	15	42	35	8	
Only US (*n* = 16)	19	50	25	6	3 Had an US follow up (at 1; 2; 4 years) prior to excision
MRI 2nd line (*n* = 6)	–	50	33	17	Subsequent MRI was < 7 months following the US, except in 1 case that was 2 years following the US
CT 2nd line (*n* = 4)	25	–	75	–	2 Had CT 2 years following the initial US 2 Had CT within 4 months of initial US and were then followed up with MRI or US
MRI (*n* = 4)	25	25	50	–	2 Had an MRI follow up (at 3 years; 2 then 4 years)
CT (*n* = 1)	–	–	100	–	Had a subsequent MRI 3 days later, followed by an US guided core Biopsy

^a^Risk factors: size > 5 cm, male sex, age > 55, history of previous mass at same site

^b^Percent of row total

Radiology reports were screened for features suggestive of malignancy, and the results are outlined in Table 3. There was a significant difference between the number of features that were (yes or no) and were not discussed (DND) in the radiology report, *X*^2^ = 28.2, *p* < 0.0001. The most common feature discussed was the estimated size of the lesion (75% of reports), however, 30% of these reports did not provide estimates for all three dimensions. Presence of calcifications, septa and nodular/globular foci were seldom mentioned. Estimated percent fat composition was never reported, although descriptors for lesion morphology were provided (e.g., high signal on T_1_ or T_2_, *homogenous* fat suppression, well defined hyperechoic ovoid density).

**Table 3:** Radiological features suggestive of malignancy discussed in the final report

	Yes (%)	No (%)	DND^a^ (%)
Size	75	0	25
Calcifications	6	8	86
Septa > 2 mm	6	0	94
Nodular/globular foci	3	19	78
% Fat composition	0	0	100

^a^Did not discuss

## Discussion

The purpose of this study was to retrospectively identify cases of head and neck lipomas that were surgically resected over a 5-year period and to identify the radiological modality chosen, features discussed in the final report, and to compare management to the ESSR guidelines. ENT and plastic surgery services made up the majority of the head and neck lipoma workload. Regions of the head and neck that received the most radiological investigation included the supraclavicular and anterior neck, which tended to be referred to ENT services; whereas lipomas of the forehead and scalp were frequently not imaged and typically referred to plastics. Twenty-two percent of all lipomas received radiological investigation, with 84% initially receiving an ultrasound, in line with ESSR guidelines [15]. Thirty-seven percent of these lipomas received subsequent imaging, the majority of which were MRI, in line with ESSR guidelines. Furthermore, radiological features suggestive of malignancy were inconsistently reported on and not documented in multidisciplinary discussions.

Differentiating benign from malignant adipocytic soft tissue tumours at the initial clinical presentation can be challenging, particularly at our institution which is the national sarcoma centre where index for suspicion is high. Sarcoma comprises < 1% of all malignancies diagnosed annually, and liposarcoma accounts for 17–30% of all soft tissue sarcomas [6]. Even though liposarcoma is one of the most common soft tissue sarcomas, it is rare. Furthermore, head and neck liposarcoma accounts for less than 5% of all liposarcoma [6]. This agrees with the incidence of head and neck liposarcoma found at this institution; 1.4% over a 5-year period. Moreover, this is likely an overestimation of the true incidence of head and neck liposarcoma, as this is a national sarcoma centre, and false positives were excluded by study design.

Twenty two percent of all lipomas received radiological investigation in this study, with 84% initially receiving an ultrasound, in line with guidelines [15]. This contrasts with El-Monem et al*.*, where CT was predominantly used. Although this study pre-dates the ESSR Guidelines, El-Monem et al*.*, pointed out the cost/benefit ratio in developing countries between CT and MRI. However, Ultrasound is also cost effective in addition to using no ionizing radiation, and therefore, should still be the first line investigation in developing countries. Thirty-seven percent of the imaged lipomas in this current study received subsequent imaging, the majority of which were MRI, in line with guidelines. According to the ESSR guidelines, MRI is the imaging modality with the best soft tissue contrast, while CT can be complementary to show calcifications or ossification. Interestingly, none of the patients that received a CT had a documented contraindication to MRI, and the CTs were typically followed up with an MRI. Additionally, CT was never performed following an MRI in any of the cases reviewed in this study. Taken together, these results suggest that guidelines are not being consistently followed at the point of imaging referral. The limited awareness and use of radiology referral guidelines is not a new problem [18], and there have been several proposed solutions including adequate integration into existing referring physician’s workflow [18], along with incorporating machine learning algorithms [19].

Specific to differentiating lipoma from liposarcoma, we additionally evaluated the use of radiological features suggestive of malignancy previously identified in the literature [11, 13], including low percent fat containing lesion, calcifications, thick septa > 2 mm, and non-lipomatous nodular or globular foci. These features were seldom included in the final report, suggesting how infrequent these features exist in benign lipoma. Although not specific to the head and neck, Wang et al. [20] published their own weighted scoring system for differentiating liposarcoma from lipoma based on clinical and MRI features, while Asano et al. [21] similarly published a scoring system based on clinical, radiological, and histopathological features. It would have been of interest to compare the scoring systems in the head and neck, as this could have better alluded to risk stratification of benign and malignant adipocytic tumours. However, there were not enough head and neck liposarcomas (*n* = 2) to evaluate the predictive value of these features in this study. As future work, it would be of interest to review the images in this study to re-classify the radiological features that were not discussed as “yes” or “no.” A significant presence of these radiological features in benign lipomas would indicate low specificity and not predictive of malignancy. Furthermore, due to the retrospective nature of the study, we were not able to quantify the radiological features that were verbally discussed at MDT but not documented in the report. While we recognize the impracticality of including these features in the radiology report of every suspected lipoma, discussion of these features is important at MDT as they can inform the clinical decision-making process, including proceeding to subsequent imaging, biopsy, and surgical approach. Interestingly, there was no difference in documentation of these features between lipomas that were and were not discussed at MDT, suggesting over investigation of lipomas at MDT including further imaging and biopsy prior to surgical resection.

**Fig. 2:**
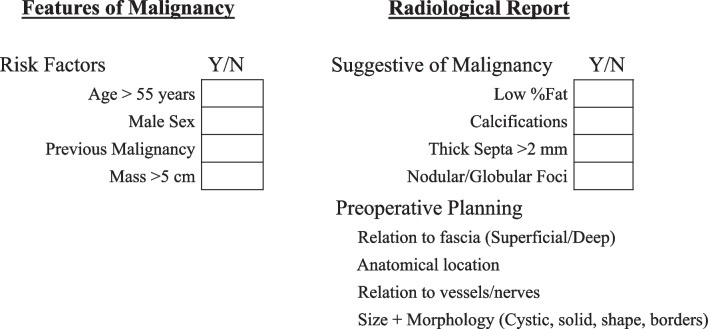
MDT Checklist for suspected Lipoma/Liposarcoma

A survey of referring clinicians and radiologists by Bosmans et al. found 97% of clinicians surveyed believed that the radiologist should know what clinical question the clinician wants answered [22]. Additionally, they found 95% of clinicians thought anyone who requests an examination that is not routine should clearly state a clinical question. Therefore, the question remains, if these radiological features of malignancy are to be included in the reports of cases that have high clinical suspicion, e.g. at MDT, should the referring clinician explicitly state this in the requisition or should the radiologists know to include them in the report? We suggest the documentation of these features for lipomas that make it to MDT through the implementation of a checklist for reporting soft tissue masses of the head and neck suspicious for malignancy, as outline in Fig. 2. This checklist could be implemented as a structured requisition or report. Checklists may have a greater role in continuing medical education by providing radiology and surgical trainees with a simple tool to aid in carrying out referrals and writing complex reports. Similarly, a recent study by Benhabib et al. [23] looked at the report reliability following implementation of Standardized reporting on preoperative CT assessment of potential living renal transplant donors and found all outcome variables to significantly improve. The authors also outlined the importance of involving the referring physicians in optimizing the standardized report template, while other studies have also highlighted the increased ease of extracting information from standardized reports [24].

## Conclusion

This study is the largest retrospective analysis in the literature of head and neck lipoma and liposarcoma to the authors' knowledge. As expected, the incidence of liposarcoma is low. There is limited awareness of radiology referral guidelines superimposed with a tendency to over-investigate lipoma. Furthermore, radiological features suggestive of malignancy were infrequently reported on and not documented in MDT discussion. Therefore, we propose an MDT checklist for referring physicians to aid in diagnostic work-up of clinically suspicious lipoma.

## Data Availability

Data can be made available upon request.

## References

[1] Choi JH, Ro JY (2021) The 2020 WHO Classification of tumors of soft tissue: selected changes and new entities. Adv Anat Pathol. 28(1):44–58 32960834 10.1097/PAP.0000000000000284

[2] Bansal A, Goyal S, Goyal A, Jana M (2021) WHO classification of soft tissue tumours 2020: an update and simplified approach for radiologists. Eur J Radiol 143:109937. 10.1016/j.ejrad.2021.109937 34547634 10.1016/j.ejrad.2021.109937

[3] Sciot R (2021) MDM2 amplified sarcomas: a literature review. Diagnostics (Basel). 11(3):496. 10.3390/diagnostics11030496 33799733 10.3390/diagnostics11030496 PMC8001728

[4] Fisher SB, Baxter KJ, Staley CA 3rd, Fisher KE, Monson DK, Murray DR et al (2013) The General Surgeon’s quandary: atypical lipomatous tumor vs lipoma, who needs a surgical oncologist? J Am Coll Surg 217(5):881–888. 10.1016/j.jamcollsurg.2013.06.003 24074812 10.1016/j.jamcollsurg.2013.06.003 PMC3805785

[5] Anderson WJ, Doyle LA (2021) Updates from the 2020 World Health Organization classification of soft tissue and bone tumours. Histopathology 78(5):644–657. 10.1111/his.14265 33438273 10.1111/his.14265

[6] de Bree E, Karatzanis A, Hunt JL, Strojan P, Rinaldo A, Takes RP et al (2015) Lipomatous tumours of the head and neck: a spectrum of biological behaviour. Eur Arch Otorhinolaryngol 272(5):1061–1077. 10.1007/s00405-014-3065-8 24800932 10.1007/s00405-014-3065-8

[7] Kale HA, Prabhu AV, Sinelnikov A, Branstetter BT (2016) Fat: friend or foe? A review of fat-containing masses within the head and neck. Br J Radiol. 89(1067):20150811. 10.1259/bjr.20150811 27542075 10.1259/bjr.20150811 PMC5124824

[8] Som PM, Scherl MP, Rao VM, Biller HF (1986) Rare presentations of ordinary lipomas of the head and neck: a review. Am J Neuroradiol 7:657–664 3088944 PMC8334668

[9] Kim KS, Yang HS (2014) Unusual locations of lipoma: differential diagnosis of head and neck mass. Aust J Gen Pract 43:867–870 25705737

[10] Najaf Y, Cartier C, Favier V, Garrel R (2019) Symptomatic head and neck lipomas. Eur Ann Otorhinolaryngol Head Neck Dis 136(2):127–129. 10.1016/j.anorl.2018.12.001 30606653 10.1016/j.anorl.2018.12.001

[11] Razek AA, Huang BY (2011) Soft tissue tumors of the head and neck: imaging-based review of the WHO classification. Radiographics 31:1923–1954 22084180 10.1148/rg.317115095

[12] Rodriguez JD, Selleck AM, Abdel Razek AAK, Huang BY (2022) Update on MR imaging of soft tissue tumors of head and neck. Magn Reson Imaging Clin N Am 30(1):151–198. 10.1016/j.mric.2021.06.019 34802577 10.1016/j.mric.2021.06.019

[13] Kransdorf MJ, Bancroft LW, Peterson JJ, Murphey MD, Foster WC, Temple HT (2002) Imaging of fatty tumors: distinction of lipoma and well-differentiated liposarcoma. Radiology 224:99–104 12091667 10.1148/radiol.2241011113

[14] ESR (2017) Summary of the proceedings of the international forum 2016: “Imaging referral guidelines and clinical decision support—how can radiologists implement imaging referral guidelines in clinical routine?”. Insights Imag 8(1):1–9. 10.1007/s13244-016-0523-4 10.1007/s13244-016-0523-4 PMC5265190 28044260

[15] Noebauer-Huhmann IM, Weber MA, Lalam RK, Trattnig S, Bohndorf K, Vanhoenacker F et al (2015) Soft tissue tumors in adults: ESSR-approved guidelines for diagnostic imaging. Semin Musculoskelet Radiol 19(5):475–482. 10.1055/s-0035-1569251 26696086 10.1055/s-0035-1569251

[16] Weiss S, Korthaus A, Baumann N, Yamamura J, Spiro AS, Lubke AM et al (2021) Musculoskeletal soft-tissue sarcoma: quality assessment of initial MRI reports shows frequent deviation from ESSR Guidelines. Diagnostics (Basel). 11(4):695. 10.3390/diagnostics11040695 33919690 10.3390/diagnostics11040695 PMC8069769

[17] Korthaus A, Weiss S, Barg A, Salamon J, Schlickewei C, Frosch KH et al (2022) Clinical routine and necessary advances in soft tissue tumor imaging based on the ESSR Guideline: initial findings. Tomography 8(3):1586–1594. 10.3390/tomography8030131 35736879 10.3390/tomography8030131 PMC9228892

[18] European Society of R (2017) Summary of the proceedings of the international forum 2016: “Imaging referral guidelines and clinical decision support - how can radiologists implement imaging referral guidelines in clinical routine?”. Insights Imag 8(1):1–9. 10.1007/s13244-016-0523-4 10.1007/s13244-016-0523-4 PMC5265190 28044260

[19] Mohammed HT, Payson LA, Gillan C, Mathews J, Diep J, Sadri-Gerrior J et al (2022) Exploring the impact of diagnostic imaging decision support embedded in an electronic referral solution on the appropriate ordering of magnetic resonance imaging for patients with knee pain: a retrospective chart review. J Eval Clin Pract 28(2):247–259. 10.1111/jep.13617 34514681 10.1111/jep.13617

[20] Wang S, Chan LWM, Tang X, Su C, Zhang C, Sun K et al (2016) A weighted scoring system to differentiate malignant liposarcomas from benign lipomas. J Orthop Surg 24(2):216–221 10.1177/1602400219 27574266

[21] Asano Y, Miwa S, Yamamoto N, Hayashi K, Takeuchi A, Igarashi K et al (2022) A scoring system combining clinical, radiological, and histopathological examinations for differential diagnosis between lipoma and atypical lipomatous tumor/well-differentiated liposarcoma. Sci Rep 12(1):237. 10.1038/s41598-021-04004-1 34997060 10.1038/s41598-021-04004-1 PMC8742117

[22] Bosmans JML, Weyler JJ, De Schepper AM, Parizel PM (2011) The radiology report as seen by radiologists and referring clinicians: results of the COVER and ROVER surveys. Radiology 259(1):184–195. 10.1148/radiol.10101045 21224423 10.1148/radiol.10101045

[23] Benhabib H, Crivellaro PS, Osman H, Gunaseelan S, Chung A, Lee JY et al (2023) Standardized reporting on the preoperative CT assessment of potential living renal transplant donors: can we create a universal report standard to meet the needs of transplant urologists? Can Assoc Radiol J. 74:269–634. 10.1177/08465371231153828 10.1177/08465371231153828 36718778

[24] Norenberg D, Sommer WH, Thasler W, D’Haese J, Rentsch M, Kolben T et al (2017) Structured reporting of rectal magnetic resonance imaging in suspected primary rectal cancer: potential benefits for surgical planning and interdisciplinary communication. Invest Radiol 52(4):232–239. 10.1097/RLI.0000000000000336 27861230 10.1097/RLI.0000000000000336

